# Compression distance can discriminate animals by genetic profile, build relationship matrices and estimate breeding values

**DOI:** 10.1186/s12711-015-0158-9

**Published:** 2015-10-13

**Authors:** Nicholas J. Hudson, Laercio Porto-Neto, James W. Kijas, Antonio Reverter

**Affiliations:** CSIRO Agriculture, Computational and Systems Biology, 306 Carmody Road, St. Lucia, Brisbane, QLD 4075 Australia

## Abstract

**Background:**

Genetic relatedness is currently estimated by a combination of traditional pedigree-based approaches (i.e. numerator relationship matrices, NRM) and, given the recent availability of molecular information, using marker genotypes (via genomic relationship matrices, GRM). To date, GRM are computed by genome-wide pair-wise SNP (single nucleotide polymorphism) correlations.

**Results:**

We describe a new estimate of genetic relatedness using the concept of normalised compression distance (NCD) that is borrowed from Information Theory. Analogous to GRM, the resultant compression relationship matrix (CRM) exploits numerical patterns in genome-wide allele order and proportion, which are known to vary systematically with relatedness. We explored properties of the CRM in two industry cattle datasets by analysing the genetic basis of yearling weight, a phenotype of moderate heritability. In both Brahman (*Bos indicus*) and Tropical Composite (*Bos taurus* by *Bos indicus*) populations, the clustering inferred by NCD was comparable to that based on SNP correlations using standard principal component analysis approaches. One of the versions of the CRM modestly increased the amount of explained genetic variance, slightly reduced the ‘missing heritability’ and tended to improve the prediction accuracy of breeding values in both populations when compared to both NRM and GRM. Finally, a sliding window-based application of the compression approach on these populations identified genomic regions influenced by introgression of taurine haplotypes.

**Conclusions:**

For these two bovine populations, CRM reduced the missing heritability and increased the amount of explained genetic variation for a moderately heritable complex trait. Given that NCD can sensitively discriminate closely related individuals, we foresee CRM having possible value for estimating breeding values in highly inbred populations.

**Electronic supplementary material:**

The online version of this article (doi:10.1186/s12711-015-0158-9) contains supplementary material, which is available to authorized users.

## Background

“*All genomes are equal*,* but some genomes are more equal than others*^1^” with apologies to George Orwell (1903–1950).

Accurate measures of genetic relationships among individuals are needed to accelerate artificial selection for genetic improvement [1] and to refine methods for gene discovery [2]. By accounting for patterns of relatedness, particularly within but also between families, relationships among individuals lay the foundation for robustly connecting genotype to phenotype. Genetic relatedness is currently estimated by traditional pedigree-based approaches (NRM for numerator relationship matrices) [3], augmented by molecular information [genomic relationship matrices (GRM)] [4]. Because meiotic recombination is stochastic and pedigree information is not always available or error free, GRM can give more precise estimates of genetic relatedness than basic pedigree information since the latter makes simplifying assumptions [4]. For example, while we predict that full-sibs and half-sibs share approximately 50 and 25 % of their DNA, respectively, simple pedigree information is unable to account for the exact percentage shared, or indeed which DNA segments have been inherited. Moreover, because of linkage disequilibrium (LD) and linkage, associations of DNA markers with quantitative trait loci (QTL) are expected to erode during successive meioses at a slower rate than pedigree relationships, which increases their utility across generations [5]. Overall, these advantages of marker-based relationships have increased the attractiveness of single nucleotide polymorphism (SNP) chips in genetic improvement programs.

GRM are essentially computed by genome-wide SNP genotype similarities (e.g. correlations) among all pair-wise combinations of individuals [4]. These correlations exploit SNP genotypes that are shared between two individuals, one SNP at a time. However, it is an open question whether correlation is the best way to relate SNP genotype data given that (1) any non-linear relationships are poorly characterised or undetected by correlations and (2) it is not immediately obvious what is the best approach when assessing whole genomes that have been abstracted to long complex numerical systems described by a small three letter SNP alphabet (0,1,2). We hypothesize that there is unexplored potential to characterise alternative and/or complementary measures of relatedness, for example through pattern recognition approaches sensitive to the information contained in complex patterns. One such alternative is normalised compression distance (NCD), which has previously been used to successfully cluster various data, including musical compositions into genres [6]. The basic principle of NCD as it applies to genomics is that patterns in the SNP genotypes from one individual can be used to describe similar patterns in the SNP genotypes from a second individual. The ability of one individual to describe another individual can be quantified mathematically by data compression, approximated via real-world compressors like the gzip application tool of UNIX systems (http://www.gzip.org). If the data compression relating the two genomes is strong, then they are deemed to be closely related and are awarded a short distance. Applying this process systematically across a genotyped population can be used to build a compression relationship matrix (CRM), analogous to a GRM. In a preliminary study, we explored the application of NCD to two sheep populations, one that included multiple breeds [7] and another with known sire groups and a half-sib population structure (unpublished data). We found that the method had merit in recovering both breed history and sire group structure.

Prior to that, we explored a basic measure of within-genome compression efficiency (CE) by expressing the SNP genotype file sizes in bits before and after data compression by the gzip tool. Plotting these CE values relative to heterozygosity [8] yielded clusters of individuals that are similar to those produced by population differentiation such as F statistics (*F*_ST_), and consistent with phylogeography. Genome-wide CE can be considered as reflecting patterns in both allele order and allele proportion that are known to differ systematically between breeds, but to be shared among closely related individuals. These shared patterns among individuals could include, but not be limited to, genome-wide heterozygosity and runs of homozygosity [9]. Similar to GRM correlation, CE is a hypothesis-free pattern recognition tool. It can exploit very complex shared patterns that do not need to be defined a priori. The utility of inferred relationship matrices can be validated in the normal manner—that is, by using them to predict genetic merit for complex phenotypes using best linear unbiased prediction (BLUP) and evaluating their accuracy.

Here, we studied two animal populations of commercial relevance to Australian agricultural production that have matching phenotype data for yearling weight, a complex phenotype of moderate heritability, one Brahman (BB) and one Tropical Composite (TC). These populations have recorded pedigrees that show the presence of both full-sib and half-sib individuals. Furthermore, these populations represent historically admixed populations that were founded from contributions of both *Bos indicus* and *Bos taurus* progenitors, which are sub-species that arose from independent domestication events and last shared a common ancestor more than 200,000 years ago [10]. For the first time, we compared compression-based best linear unbiased predictions (CBLUP) with genomic (GBLUP) and pedigree-based (PBLUP) predictions for yearling weight. We present the outcome of the clustering, the proportion of missing heritability [11] and the prediction accuracies for yearling weight that were obtained by using different approaches to estimate the relationship between individuals.

## Methods

### Animal resources and SNP genotyping platforms

Animals, phenotypes and genotypes used in this study were a subset of those used in [12]. Briefly, we used data on 816 Brahman (BB) and 1028 Tropical Composite (TC) cows genotyped using either the BovineSNP50 [13] or the BovineHD (Illumina Inc., San Diego, CA, USA) that includes more than 770,000 SNPs. For animals that were genotyped with the lower density array, genotypes were imputed to higher-density based on the genotypes of relatives based on pedigree, as described previously [14]. The imputation was performed using 30 iterations of BEAGLE [15] within breeds, using 519 Brahman and 351 Tropical Composite animals genotyped using the BovineHD as reference. From the resulting 729,068 SNP genotypes per individual, we extracted the genotypes from 71,726 SNPs that were highly polymorphic in *Bos indicus* cattle (GGP Indicus HD Chip; http://www.neogeneurope.com/Agrigenomics/pdf/Slicks/NE_GeneSeekCustomChipFlyer.pdf).

### Animal clustering by genotype

#### Genome-wide CE

First, we computed animal-to-animal relationships that could be ascertained from genotype data using the basic CE approach corrected for heterozygosity (CEh), as described in [8]. This approach computes the CE for the genotype file of each individual and then expresses it against heterozygosity (*Het*) across the whole genome:

$${\text{CEh}}\; = \;{{\left( {\frac{{{\text{S}}_{\text{B}} - {\text{S}}_{\text{A}} }}{{{\text{S}}_{\text{B}} }}} \right)} \mathord{\left/ {\vphantom {{\left( {\frac{{{\text{S}}_{\text{B}} - {\text{S}}_{\text{A}} }}{{{\text{S}}_{\text{B}} }}} \right)} {Het}}} \right. \kern-0pt} {Het}},$$

where S_B_ and S_A_ indicate the genotype file size expressed in bits before and after compression by gzip, respectively. The underlying principle of CEh is the same as for any genetic clustering method: the closer the match in the numerical patterns present in two genotype files is, the closer is the inferred genetic relationship between them. No attempt was made to discriminate DNA segments that were identical by descent from those that were identical by state.

#### Normalised compression distance computation

The main weakness of CEh is that two genotype strings can have the same CE and heterozygosity despite being different (e.g. 0000000000 and 2222222222). It is not clear how common this phenomenon is in real population genetic data, but it has the potential to confound some of the observed clustering. To address this, we used normalised compression distance (NCD) [6] to develop alternative measures of relationship. NCD is a way of measuring the similarity between two objects. NCD is obtained by approximating a non-computable similarity metric called normalized information distance (NID). The principle is that NCD will award short distances to highly related sequences, on the grounds that shared patterns result in compression gain when two similar files are concatenated, but not when two dissimilar files are concatenated. In other words, a short distance is awarded when the information in the first genotype file can be used to describe the information in the second genotype file. We used the gzip application tool of UNIX systems (http://www.gzip.org) as our real world compressor. The application gzip is based on the lossless data compression algorithm DEFLATE that was originally described by [16]. NCD only clusters those genotype files that compress the same for the same reasons. That is, in contrast to CEh, 0000000000 will now only cluster with 0000000000 and not with 2222222222.

As previously reported [7], the formula for the computation of NCD between two individuals *x* and *y* based on their respective SNP genotype sequence is:

$${\text{NCD}}\left( {x,y} \right) = \frac{{Z\left( {xy} \right) - { \hbox{min} }\left\{ Z\left( x \right),Z\left( y \right)\right\} }}{{{ \hbox{max} }\left\{ Z\left( x \right),Z\left( y \right)\right\} }},$$

where *Z*(*xy*) represents the size of the compressed file that contains both SNP genotype sequences to be compared and *Z*(*x*) and *Z*(*y*) are the sizes of the compressed file with the separate SNP genotypes for *x* and *y*, respectively.

#### Building a compression relationship matrix from the NCD values

Relationship matrices are based on estimates of similarity, but through NCD we have computed ‘distance’ not similarity. Therefore, the construction of the CRM from all pair-wise NCD values first requires conversion of the compression distance to an equivalent similarity measure. While distance and similarity share commonalities they are not equivalent. In practice, there are numerous ways of inter-relating them. Here, we explored two approaches, producing two different CRM (CRM1 and CRM2) from the same NCD input.

The first method (CRM1) made use of the universal distance (*d*) to similarity (*s*) conversion law of Shepard [17]. We used $s_{i,j} = 2.5e^{{ - 5d_{i,j} }}$, which was selected in an ad hoc fashion by confirming that the resulting similarity between individuals *i* and *j* (*s*_*i,j*_) covered the 0–1 interval observed for correlation (and therefore for all GRM), where *d*_*i,j*_ is the NCD between the *i* and *j* individual pair. This method proved to have a scaling issue which can bias estimates of genetic parameters. This problem was overcome through computation of CRM2 (described below).

The second method (CRM2) attempted to better ground the NCD in established genetics—that is, an expectation of relatedness of 1 for self–self pairs, 0.5 for full sibs and 0.25 for half-sibs. This expectation is governed by the laws of inheritance and the likely molecular outcomes of meiosis when applied to a diploid mammalian genome. CRM2 used a linear conversion method defined as follows:

$$s_{i,j} \left\{ \begin{array}{ll} \frac{{{\text{Mean}}(d_{i,j} )}}{{d_{i,j} }} & {\rm for} \quad i = j \hfill \\ 1.75\left[ {1 - \frac{{d_{i,j} - {\text{Min}}(d_{i,j} )}}{{{\text{Max}}(d_{i,j} ) - {\text{Min}}(d_{i,j} )}}} \right] & {\rm for} \quad i \ne j \hfill \\ \end{array} \right.\;.\;$$

This linear method has the appealing feature of yielding an average value of 1 for self–self pairs, including a spread around 1 that reflects inbreeding. This result shows that the CRM2 matrix is scaled in a manner more suitable for the estimation of genetic parameters. The remaining values approximate 0.5 for full sibs, 0.25 for half-sibs and so on. Similar to GRM but unlike NRM, these values were not pre-defined by pedigree expectation but derived from the SNP genotype data. Therefore, they are likely to more accurately capture the observed variability that is inherent to the process of meiosis that gives rise to each individual compared to its relatives.

The third compression-based relationship matrix (CRM3) was entirely independent of the NCD approach described above. The aim of CRM3 was to produce another set of genetically sensible relatedness values, through application of a two-step process: an initial window-based CE step and a subsequent correlation step. To achieve this, we produced a matrix with as many columns as sets of 50 consecutive SNPs that can be built from the genotype file, and as many rows as animals in the analysis. Individual cells in this matrix contain the CE value for each window for each animal. This matrix was then used as input for a correlation analysis so that, for each pair of animals, the correlation across their respective CE values was used as a measure of relatedness. Animals that had sets of SNPs that compressed in the same way along their respective genomes were awarded high correlations.

#### GRM computation

The construction of the GRM was based on the correlation between genotypes and was computed according to the methodology developed in [4]:

$${\text{GRM}} = \frac{{{\textbf{ZZ}}^{T} }}{{2\mathop \sum \nolimits p_{i} (1 - p_{i} )}},$$

where **Z** is the (number of animals by number of SNPs) matrix of genotypes and *p*_*i*_ is the frequency in the population of the B allele for the *i*th SNP. **ZZ**^T^ represents the number of shared SNP alleles between all pairs of individuals and the division of **ZZ**^T^ by $2\mathop \sum \nolimits p_{i} (1 - p_{i} )$ aims at scaling the GRM to make it analogous to the numerator relationship matrix (NRM) based on pedigree. This is the standard approach for genomic prediction of breeding values [4].

**Fig. 1:**
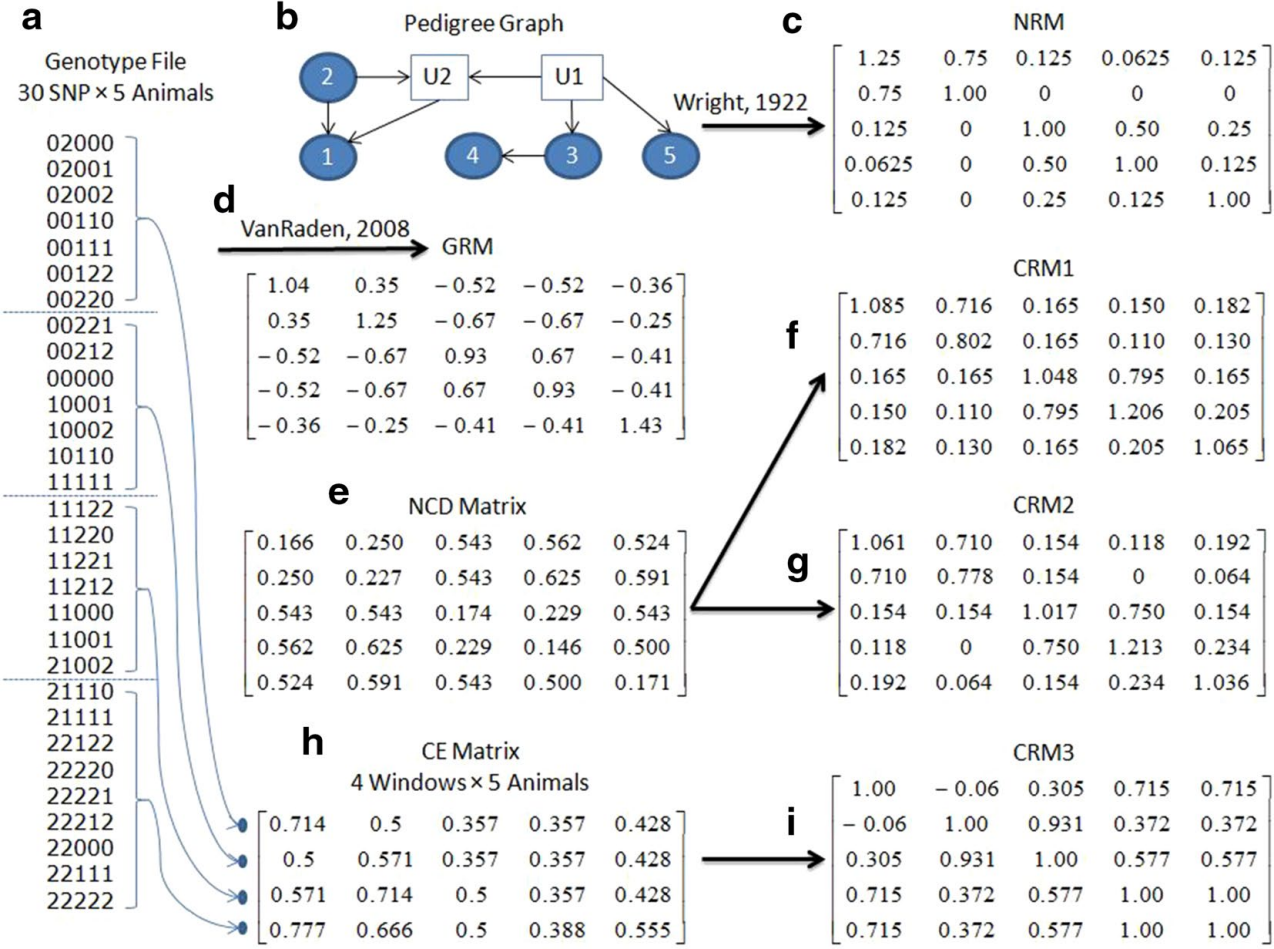
Given a genotype file (**a**) and a plausible pedigree (**b**), one can compute an NRM (**c**) and a GRM (**d**). One can also compute an NCD matrix (**e**) which in turn can be transformed into CRM1 (**f**) and CRM2 (**g**) given two different distance to similarity transformations. A sliding window-based version of the CE analysis (**h**) can be used to generate a correlation matrix which underpins the computation of CRM3 (**i**)

The computations of NRM, GRM, CRM1, CRM2 and CRM3 are schematically summarised in Fig. 1 using toy examples. The UNIX scripts for these examples are in Additional file 1. We begin with a genotype file (a in Fig. 1) that comprises 30 SNPs and five animals. The genotype profiles of Animals 1, 2, 3, 4 and 5 are encrypted by {10 “0” + 10 “1” + 10 “2”}, {3 “2” + 10 “0” + 10 “1” + 7 “2”}, {3 * {3 “0” + 3 “1” + 3 “2”} + “0,1,2”}, {3* {3 “0” + 2 “1” + 3 “2” + “1”} + “0,1,2”}, and {10 * “0,1,2”}, respectively. It becomes immediately apparent that the profiles of Animals 1 and 2 and those of Animals 3 and 4 are very similar. On closer inspection, we also find a similarity between the profiles of Animals 3 and 5 because each trio of identical genotypes (e.g. “000”) in Animal 3 is matched by a trio of “0,1,2” in Animal 5.

The encrypted genotype profiles are awarded the following genome-wide CE; 30, 27, 23, 20 and 32 %, respectively for Animals 1 to 5. On the one hand, Animals 1 and 5 have the most regular genotypes, resulting in the highest CE and on the other hand, Animals 3 and 4 have the most irregular genotypes, resulting in the lowest CE. A plausible pedigree graph is given in b of Fig. 1, which also contains two un-genotyped animals, U1 and U2, and where arrows indicate direction from parent to offspring. Based on this pedigree and these genotypes, we can compute the NRM (c in Fig. 1) and the GRM (d in Fig. 1), respectively.

The NRM reveals that Animal 1 is the sole inbred animal since it results from a parent-offspring mating. The GRM reveals that Animals 2 and 4 are the most inbred and captures the strong (parent-offspring) relationship between Animals 1 and 2 (GRM value = 0.35) and Animals 3 and 4 (GRM value = 0.67). The GRM also captures the most distant relationships (GRM value = −0.67), i.e. of Animal 2 with Animals 3 and 4, which are unrelated based on the NRM.

Based on this genotype file, we can also compute the NCD matrix (e in Fig. 1), which shows the shortest distances on the diagonals (i.e. self–self comparisons). Consistent with the GRM and the NRM, the longest distances observed in the NCD matrix (NCD values = 0.625 and 0.591) are between Animal 2 and Animals 3 and 4. Two distance-to-similarity transformations were used to generate CRM1 (f in Fig. 1) and CRM2 (g in Fig. 1). These two CRM differ in that CRM1 has un-scaled diagonal values and unbounded off-diagonal values, whereas CRM2 has scaled diagonal values such that they average to 1 and off-diagonal values bounded between 0 and 0.75.

The genotype file can also be divided in windows (or genomic regions) of consecutive SNPs and CE can then be computed for each window by animal combination. Here, we have four windows of 7, 7, 7, and 9 consecutive SNPs giving rise to the CE matrix h of Fig. 1. Windows with seven identical genotypes, such as the first window in Animal 1 and the third window in Animal 2, yield a high CE of 71.4 %. Column-wise, a correlation matrix based on CE values for all pairs of animals is computed to generate CRM3 (i in Fig. 1). In this case, Animals 4 and 5 are identical according to CRM3, while the relationship between Animals 1 and 2 has disappeared.

### Comparing CRM and GRM

#### Variance components estimates

We used mixed-model equations and the Qxpak.5 software [18] for the estimation of genetic parameters and the prediction of breeding values for yearling weight in the two populations. The general model was as follows:

$${\mathbf{y}} = {\mathbf{X}}{\boldsymbol{\upbeta}}\, + \,\sum\limits_{r} {{\mathbf{Zu}}{}_{r}} + \,{\mathbf{e}},$$

where **y** is the vector of yearling weight observations, **X** in an incidence matrix relating observation in **y** with the vector of fixed effects in **β** (i.e., contemporary group comprised of sex, year and location, and the covariates of age of dam, indicine percent and age of measurement) [12]. The summation goes for *r*, the number of random components fitted in the model. **Z** is an incidence matrix relating observations in **y** with the vector of random additive effects in **u**_*r*_ which are assumed to be normally distributed with zero mean and variance ${\text{V}}\left( {{\mathbf{u}}_{r} } \right) = {\mathbf{C}}_{r} \sigma_{{{\text{u}}r}}^{2}$, where **C**_*r*_ is the relationship matrix based on either the pedigree (NRM) or markers (GRM, CRM1, CRM2 or CRM3), and $\sigma_{{{\text{u}}r}}^{2}$ is the additive genetic variance associated with **u**_*r*_. Finally, **e** is the vector of random residual effects assumed to be normally distributed with zero mean and variance ${\text{V}}\left( {\mathbf{e}} \right) = {\mathbf{I}}\sigma_{\text{e}}^{2}$, where **I** denotes an identity matrix and $\sigma_{\text{e}}^{2}$ is the residual variance.

Twelve models were explored: one each (i.e., four) with a single additive effect from either relationship matrix (NRM, GRM, CRM1 and CRM2), and then an informative subset of combinations of the above models. These 12 models are defined in Tables 1 and 2 for BB and TC, respectively.

**Table 1:** Estimates of variance components for BB cattle: comparison of estimates based on pedigree (NRM), normalized compression distance (CRM1 and CRM2) and genomic relationships (GRM)

Model effects	Ve	Vn	Vg	Vc1	Vc2	Vp
1. NRM	172.3642	174.8665				347.2307
2. GRM	167.3202		179.9430			347.2632
3. CRM1	161.9650			121.9581		283.9231
4. CRM2	168.6462				195.9485	364.5947
5. NRM + GRM	115.5917	113.9303	129.9348			359.4568
6. NRM + CRM1	106.5055	123.3128		78.3912		308.2095
7. NRM + CRM2	113.3747	118.5415			140.8651	372.7813
8. GRM + CRM1	102.9185		130.3848	75.1383		308.4416
9. GRM + CRM2	151.5553		103.5573		104.4451	359.5577
10. CRM1 + CRM2	103.7444			76.7594	140.8459	321.3497
11. NRM + GRM + CRM1	79.1332	88.9443	99.2563	57.8128		325.1466
12. NRM + GRM + CRM2	82.3409	92.4328	98.8974		105.2992	378.9703

*Ve* residual variance, *Vn* genetic variance based on pedigree NRM, *Vg* genetic variance based on the genotype GRM; *Vc1* genetic variance based on the genotype CRM1, *Vc2* genetic variance based on the genotype CRM2, *Vp* phenotypic variance

**Table 2:** Estimates of variance components for TC cattle: Comparison between pedigree (NRM), normalized compression distance (CRM1 and CRM2) and genomic relationship (GRM)

Model effects	Ve	Vn	Vg	Vc1	Vc2	Vp
1. NRM	220.1370	207.5774				427.714
2. GRM	217.4226		212.8462			430.269
3. CRM1	195.7910			155.4020		351.193
4. CRM2	223.5068				222.1801	445.686
5. NRM + GRM	143.1428	143.7108	159.4054			446.259
6. NRM + CRM1	131.0090	149.6712		98.5048		379.185
7. NRM + CRM2	146.4022	146.6484			165.8576	458.908
8. GRM + CRM1	127.3081		159.2306	94.8720		381.411
9. GRM + CRM2	208.1053		120.8427		110.7054	439.653
10. CRM1 + CRM2	129.0538			97.1270	168.0027	394.183
11. NRM + GRM + CRM1	97.5807	109.4418	122.6173	72.4005		402.040
12. NRM + GRM + CRM2	105.9689	116.9839	122.6360		125.9694	471.558

*Ve* residual variance, *Vn* genetic variance based on the pedigree NRM, *Vg* genetic variance based on the genotype GRM, *Vc1* genetic variance based on the genotype CRM1, *Vc2* genetic variance based on the genotype CRM2, *Vp* phenotypic variance

Using yearling weight, we compared the performances of NRM, GRM, CRM1, CRM2 and CRM3 according to the resultant genetic parameters, EBV and prediction accuracies. For the computation of accuracy, 20 % of phenotypes were randomly set to missing values. The reported accuracy was the average of 20 random splits of the data (i.e. 80 % calibration versus 20 % validation). Estimates of breeding values based on NRM, GRM, CRM1, CRM2 and CRM3 were compared and the accuracy of the resulting predictions was computed from the correlation between the EBV and the adjusted phenotypes (Table 3).

**Table 3:** Accuracy of estimates of breeding values from a model with a single random additive effect derived using different relationship matrices

Relationship matrix	BB	TC
NRM	0.182 (0.091)	0.172 (0.043)
GRM	0.228 (0.091)	0.163 (0.052)
CRM1	0.216 (0.085)	0.167 (0.045)
CRM2	0.232 (0.095)	0.172 (0.046)
CRM3	0.167 (0.066)	0.042 (0.026)

Means with standard deviations in brackets of 20 iterations in each of which a random 20 % of the observations was set to missing values and predicted from the remaining 80 %

Models 5, 6 and 7 (i.e. GRM, CRM1 and CRM2) can estimate the fraction of missing heritability (*C*_miss_) using the formulae of [11]:

$$C_{miss} = 1 - \frac{{\sigma_{u}^{2} }}{{\sigma_{a}^{2} + \sigma_{u}^{2} }},$$

where $\sigma_{u}^{2}$ is the variance due to the genotype data (i.e. either GRM or CRM1 or CRM2 in our context) and $\sigma_{a}^{2}$ is the estimate of the additive genetic variance based on pedigree (i.e. the NRM in our context). NRM, GRM, CRM2 and CRM3 have scaled values with self–self pairs close to or equal to 1. This implies that any differences between their genetic parameter estimates are unlikely to be a simple artefact of scaling.

#### Signatures of selection

In order to detect signatures of selection and regions of evolutionary interest, we applied a sliding window version of CE, as previously described in [8]. This approach exploits the sensitive pattern recognition capability of CE to identify haplotype blocks that occur in one population but not in the other. Briefly, the CE of non-overlapping windows at the population level was computed for both populations and corrected for heterozygosity (CEh). The correction for heterozygosity de-emphasizes simple patterns that are enforced by runs of homozygosity (ROH). We computed 1435 windows of 50 consecutive SNPs across the 71 K SNPs. For comparison purposes, the *F*_ST_ [19] in the BB vs. TC contrast was computed for each SNP. Then, the *F*_ST_ of the whole window was estimated based on the average *F*_ST_ of all SNPs contained in the window.

## Results

### Clustering animals by genotype

**Fig. 2:**
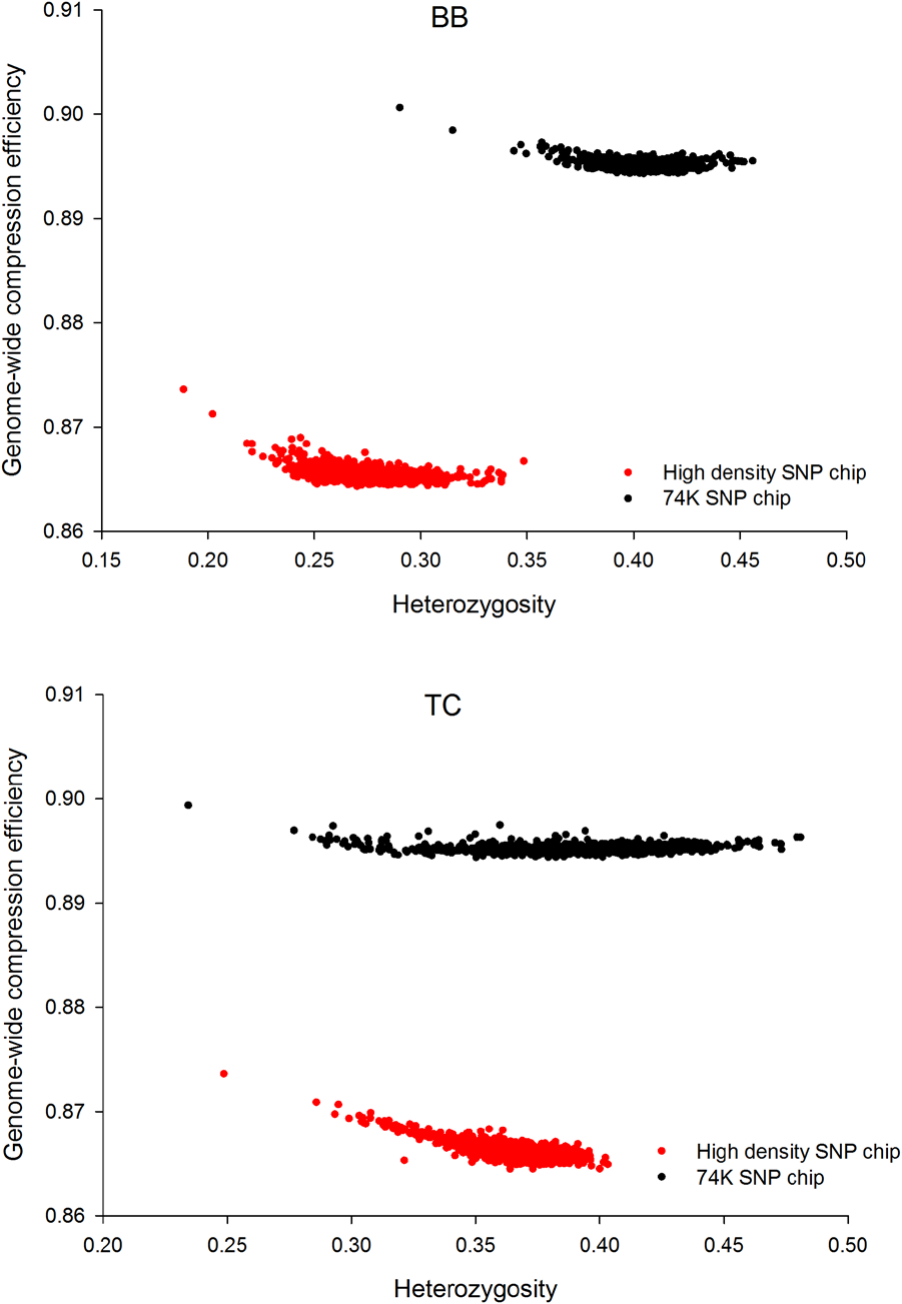
Comparison of CEh using different genotyping platforms. A comparison of CEh for BB (*top panel*) and TC (*bottom panel*) cows genotyped using both the HD chip (*red dots*) with 750 K SNPs and the new 71 K Indicus SNP chip (*black dots*). Each point represents a single animal

In Fig. 2, each point in the scatter represents either a single BB (top panel) or TC (bottom panel) animal. Two animals that cluster together can be assumed to share more genotype patterns than two animals that are further apart and thus are more likely to be related by descent. The consequence of using the new Indicine SNP chip was higher heterozygosity (particularly in the BB population), coupled with greater CE when compared to genotyping the same population using the Bovine HD BeadChip. It is likely that the increase in heterozygosity observed using the Indicine SNP chip resulted from a reduction in SNP ascertainment bias [20] that is associated with genotyping *Bos indicus* populations using the HD BeadChip that was designed for *Bos taurus*. It is less clear why the CE also increased but this may reflect the Indicine SNP chip’s greater ability to exploit regularities at common runs of heterozygous sites. Like any other form of regularity, runs of heterozygosity (strings of 1s) are a possible source of compressible patterns in genomes with high heterozygosity. Based on its improved performance, all remaining analyses were performed using the Indicine SNP chip.

#### Relatedness between animals using NRM, GRM and NCD

Tables 4, 5 and 6 show summary data that relate NRM with GRM, NCD and CRM. The pedigree-based NRM [1] was computed recursively after tracing back three generations of ancestors. No inbreeding was detected based on pedigree and the self–self relationships for the 816 BB individuals averaged at close to 1 for GRM, CRM2 and CRM3 (Table 6).

**Table 4:** Summary statistics for BB cows compared using NRM, GRM and NCD

NRM	N	GRM				NCD			
		Mean	SD	Min	Max	Mean	SD	Min	Max
0.0625	50	0.044	0.026	−0.006	0.111	1.050	0.011	1.025	1.071
0.1250	768	0.109	0.025	0.028	0.195	1.027	0.012	0.988	1.071
0.2500	8724	0.196	0.086	−0.108	0.386	0.997	0.033	0.904	1.117
0.3125	90	0.281	0.034	0.215	0.369	0.934	0.019	0.889	0.978
0.5000	201	0.288	0.068	0.167	0.473	0.957	0.034	0.782	1.007
1.0000	816	0.996	0.039	0.928	1.670	0.118	0.002	0.112	0.123

**Table 5:** Summary statistics for TC cows compared using NRM, GRM and NCD

NRM	N	GRM				NCD			
		Mean	SD	Min	Max	Mean	SD	Min	Max
0.03125	832	0.004	0.032	−0.077	0.158	1.075	0.011	1.038	1.098
0.06250	2659	0.038	0.048	−0.137	0.172	1.052	0.014	0.986	1.095
0.12500	630	0.061	0.037	−0.049	0.149	1.025	0.020	0.966	1.072
0.25000	15190	0.201	0.092	−0.075	0.455	0.987	0.046	0.799	1.100
0.31250	316	0.066	0.028	−0.003	0.157	1.051	0.009	1.023	1.074
0.50000	683	0.229	0.042	0.103	0.509	0.994	0.023	0.764	1.032
1.00000	1028	1.000	0.056	0.874	1.401	0.118	0.002	0.113	0.124

**Table 6:** Summary statistics for self–self pairs in both populations using NRM, GRM, CRM1, CRM2 and CRM3

	Brahman (N = 816)			Tropical composite (N = 1028)		
	Mean	Min.	Max.	Mean	Min.	Max.
NRM	1.000	1.000	1.000	1.000	1.000	1.000
GRM	0.995	0.928	1.257	1.000	0.874	1.401
CRM1	1.387	1.351	1.425	1.388	1.345	1.423
CRM2	1.000	0.958	1.049	1.000	0.950	1.045
CRM3	1.000	1.000	1.000	1.000	1.000	1.000

For relationships corresponding to NRM values of 0.25 (i.e. those existing between half-sibs or between grand-parent and grand-offspring), the average GRM were equal to 0.196 and 0.201 for BB and TC cattle, respectively. Self–self relationships were equal to 0.997 and 0.987 for BB and TC cattle, respectively.

**Fig. 3:**
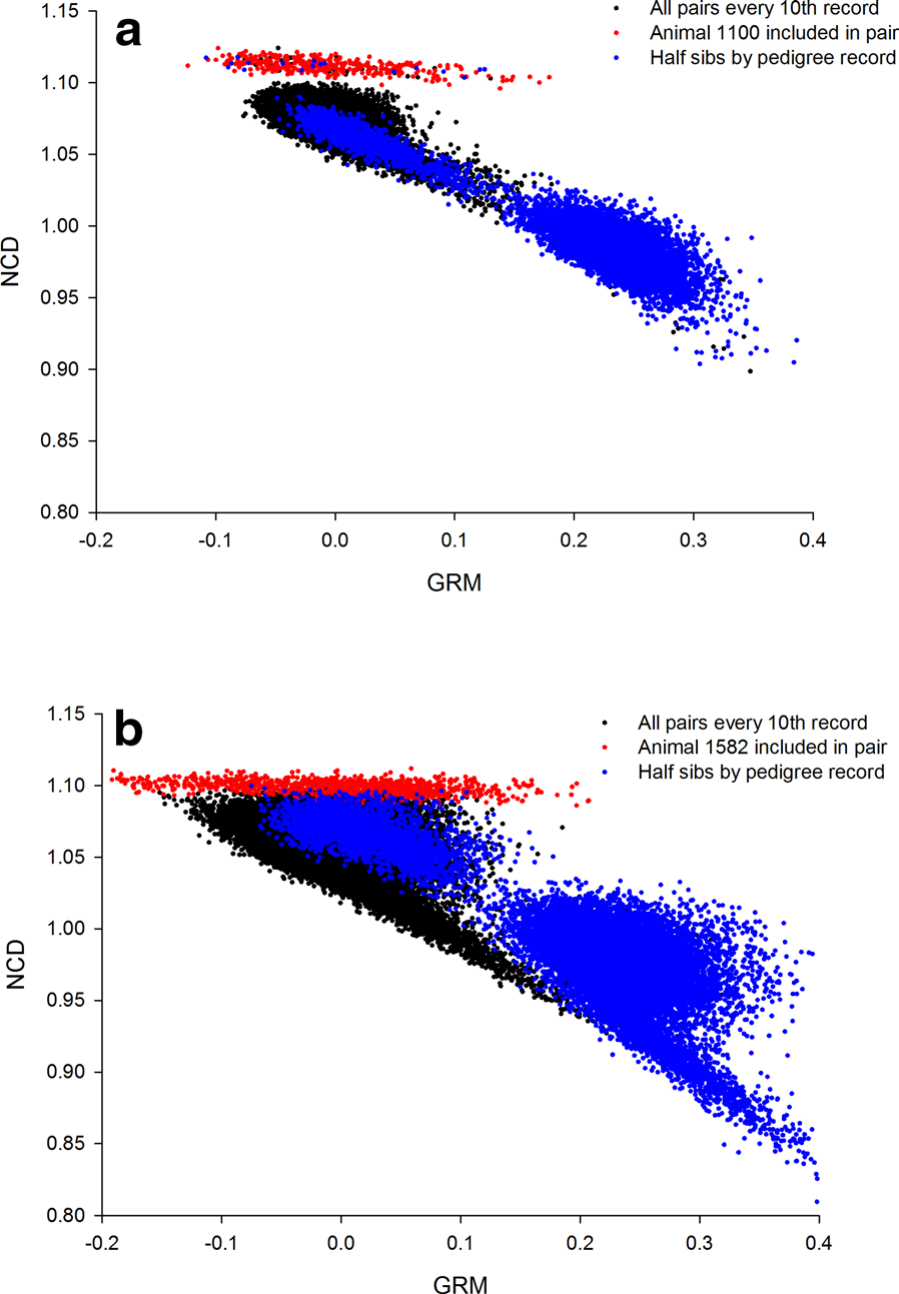
Comparison of GRM and NCD. Each point represents a pair of BB (BB; **a**) and TC cows (TC; **b**). We have plotted the parameter space showing all pairs except the self–self pairs. An outlier animal with an unusual Zebu contribution was identified by NCD but not by GRM for both populations (*red dots*). Half-sibs in a pedigree form a main cluster around the expected GRM of 0.25

The relationship between GRM and NCD for each pair of individuals is plotted in Fig. 3 for the main bulk of the data. The full parameter space including self–self pairs is in Additional file 2: Figure S1. Figure 3 reveals a population sub-structure in both populations that is more complex than that obtained by analysing either GRM or NCD, separately. This suggests that these two metrics operate synergistically and together provide a more complete understanding of relationships in the population. Highlighting half-sibs (as defined by pedigree information) yielded a cluster that was centred on a GRM of 0.25. Half-sibs that had a GRM of ~0 probably represent pedigree errors or mistakes in DNA handling.

A distinct cluster with an increased NCD was observed in the BB population (Fig. 3). This was also observed for the TC animals but was less pronounced. In both populations, the pairs in these clusters had a given individual in common. For the BB population, the Zebu genetic contribution of this individual (#1100) was much smaller (i.e. 0.528 compared to greater than 0.829 for all the other animals in the population), while for the TC population, the Zebu genetic contribution of the individual in question (#1582) was substantially larger (i.e. 0.652 compared to less than 0.56 for all the other animals in the population). Surprisingly, GRM was not able to identify the markedly different Zebu contribution of these individuals in either population.

Next, we attempted to identify genome properties that were responsible for the similarities and differences between the GRM and NCD measures of relatedness. To answer this question, we overlaid the average Zebu contribution (based on a principal component analysis that also included Angus and Nelore data) [12] of each pair (see Additional file 3: Figure S2). It is clear that, for both populations, GRM and NCD resulted in measures of relationships that were much more similar to each other for pairs of individuals that were relatively purebred than for cross-bred animals. Thus, for the BB population, the linear part of the plot is enriched with BB pairs that have an average Zebu contribution of more than 0.96, while for the TC population, it is enriched in TC pairs that have an average Zebu contribution of less than 0.1. Thus, the correlation-based (GRM) and compression-based (CRM2) measures of genetic similarity tend to agree on estimates of relatedness for purebred pairs of individuals.

**Fig. 4:**
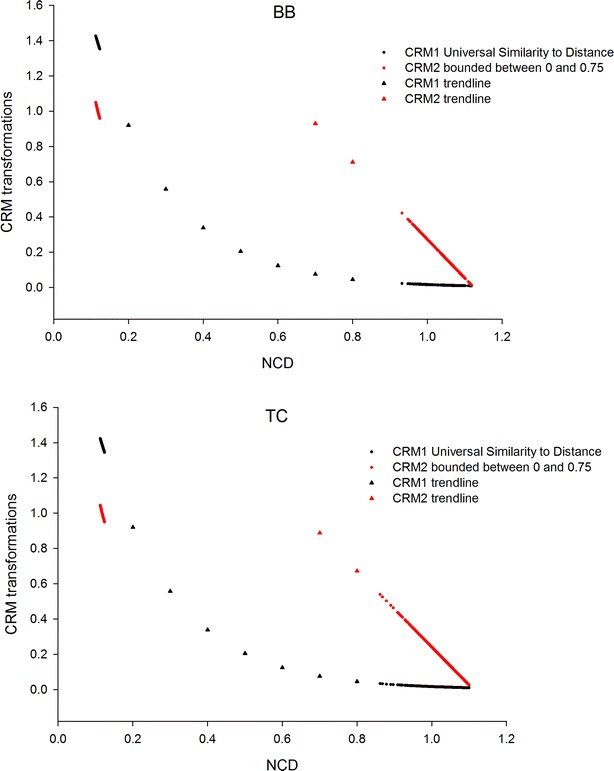
Relationship between NCD and CRM1 (*black*) and NCD and CRM2 (*red*). BB population on *top panel*, TC population on *bottom panel*. CRM1 bears a quadratic relationship to NCD, whereas CRM2 has a linear relationship. *Trend lines* were added to illustrate the difference between CRM1 and CRM2

The impact of the two NCD mapping approaches (CRM1 and CRM2) on the estimate of similarity from the same NCD value is in Fig. 4. Both versions of CRM were negatively related to NCD, because similarity is inversely related to distance. CRM2 resulted in a linear relationship whereas CRM1 resulted in a non-linear exponential relationship. The linear relationship for CRM2 explains its higher accuracy in computing genetic parameters. This is because the similarity values are more consistent with biological expectation. In other words, the similarities produced by CRM2 resemble more closely the expected genetic relationships between self–self pairs (1), full-sibs (0.5), half-sibs (0.25) and other relatives. Consequently, CRM2 can be expected to estimate genetic parameters more accurately.

#### Computational performance

We explored the computing time required for each population. For the BB population and once the relationship matrices have been built, the mixed model that contained a single additive effect took 31 s for the NRM, 4 min and 6 s for the GRM and 4 min and 10 s for the CRM2. The longer time taken for the models with either GRM or CRM2 reflects the higher density of these marker-based relationship matrices. However, the computing of NCD, which is required for CRM1 and CRM2, consumed substantially more time than the pair-wise correlations required for GRM. In our implementation, it took 102.6 and 162.4 h to compute the NCD matrices for BB and TC, respectively. The reason being the DEFLATE algorithm included in the gzip tool was not incorporated into the UNIX script, so we had to resort to numerous input and output operations that call on the compression function of GZIP externally.

Computational demands aside, the relationship matrices based on NCD were highly related to the GRM based on the strong negative correlation between pairs of individuals (high correlations corresponded to small NCD distances) (Tables 4, 5). Furthermore, we have previously documented the broad similarity in clustering produced by the GRM and NCD dimension reduction plots for sheep breeds [7].

#### Estimating genetic parameters and accuracies of predicted breeding values

Overall, estimates of genetic parameters were quite similar for GRM and CRM2 (Tables 1, 2). It should be noted that the point estimate for CRM2 explained more genetic variation (Vc2) than the NRM and GRM for both the BB and TC populations, although the absolute difference was small and likely not statistically significant. Furthermore, in the light of the equivalent cross-validation accuracies (perhaps even better for the BB population) that were obtained by using CRM2 (Table 3), it is tempting to speculate that implementing CRM2 for these populations and for this phenotype (YWT, yearling weight) using the latest Indicus 71 K SNP chip might lead to slightly better breeding decisions. Estimates of variance components based on CRM3 are in Table 7. CRM3 did not result in as much as an increase in the genetic variance explained compared to NRM and GRM as observed for CRM1 and CRM2. For both populations and in agreement with the accuracy results, the missing heritability was lowest for CRM2, which implies that it outperformed the GRM by a small margin (Table 8).

**Table 7:** Estimates of variance components with models that include CRM3

Breed	Model effects	Ve	Vn	Vg	Vc3	Vp
BB	CRM3	261.6966	–	–	83.9794	345.676
	NRM + CRM3	149.0205	142.6249	–	64.2163	355.862
	GRM + CRM3	148.1431	–	153.8330	52.7322	354.708
TC	CRM3	326.7811	–	–	109.0720	435.853
	NRM + CRM3	182.0812	183.6592	–	76.0604	441.801
	GRM + CRM3	189.8195	–	185.4988	67.5526	442.871

**Table 8:** Fraction of missing heritability for each model and population

Model effects	BB	TC
NRM + GRM	0.467	0.474
NRM + CRM1	0.611	0.603
NRM + CRM2	0.457	0.469
NRM + CRM3	0.689	0.707

As described by Roman-Ponce et al. [11], we estimated the missing heritability as the proportion of the genetic variance not captured by the marker-based relationship matrix, where the later was either the GRM or one of the three alternate CRM

#### Signatures of selection

The comparison between *F*_ST_ and CEh revealed a high positive relationship (r = 0.75), which confirmed the relevance of using CE to detect putative selected genomic regions in cattle. Genomic windows on the X chromosome consistently displayed high values for both metrics, which confirms previous results observed for populations that include *Bos indicus* and *Bos taurus* ancestry [21]. A likely explanation is that the level of sequence divergence between the X chromosomes of these two sub-species is greater than that between autosomal chromosomes [21].

**Fig. 5:**
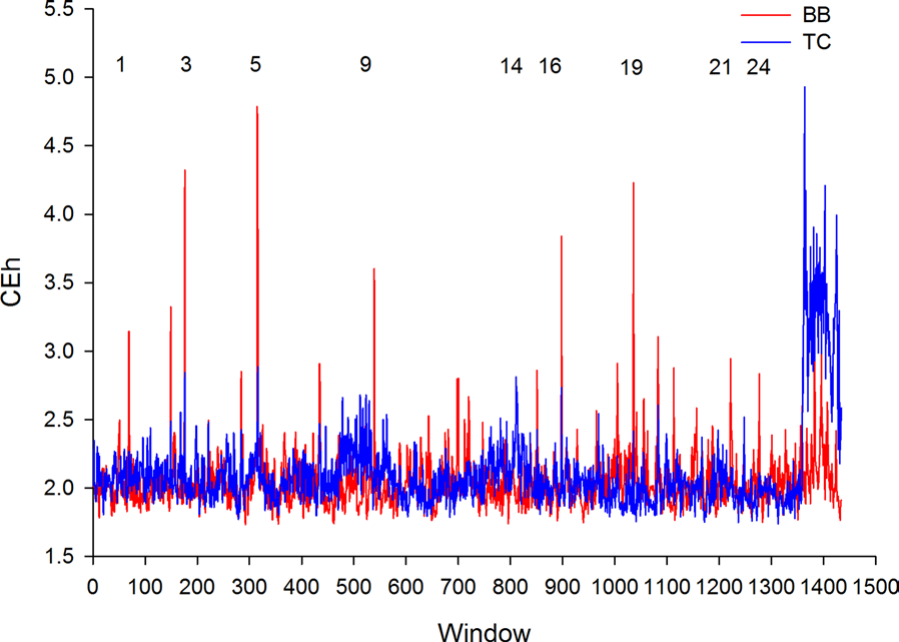
Genome-wide view of compression peaks in BB (*red* profile) versus TC cows (*blue* profile). Heterozygosity corrected CE (CEh) was estimated separately for BB (*blue*) and TC cattle (*red*) and plotted in genomic order. Windows with extreme population differences in CEh are identified in Table 9. Chromosome number is indicated above the plot

**Fig. 6:**
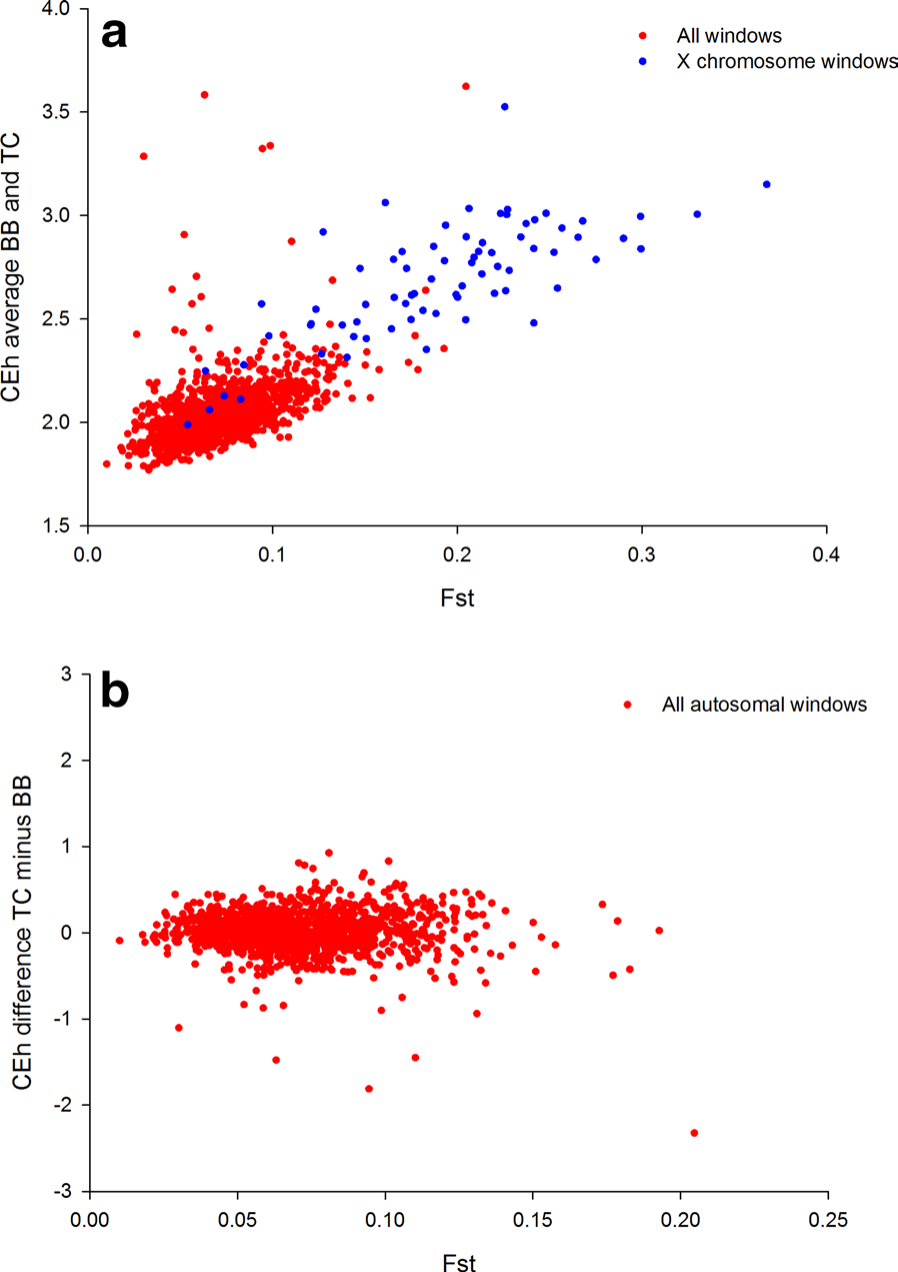
**a** Relationship between average CEh and *F*_ST_. *Each dot* represents a window of 100 consecutive SNPs. Windows in the *top left* quadrant are identified as important by CEh but not *F*_ST_. *Blue dots* are windows on the X chromosome. Windows in the *top left* quadrant are identified as important by CEh but not *F*_ST_. **b** Relationship of the difference in CEh between populations (TC-BB) with *F*_ST_

To identify autosomal genomic windows with markedly different genetic patterns between BB and TC cattle, we examined the genome-wide distribution of CEh in each population (Fig. 5). The average CEh for BB and TC populations was plotted against *F*_ST_ and yielded a positive relationship (Fig. 6a), which suggests that the approach has merit in population differentiation. The difference between the CEh for the BB and TC populations was plotted against *F*_ST_ (Fig. 6b) to identify population-specific outliers. Detailed investigation of the extreme windows in Fig. 6b allowed us to identify population-specific sweeps. The autosomal windows with the most extreme differences between the two populations are in Table 9. Nine of the ten regions had high CEh values for the BB population and much lower values for the TC population (Table 9). The gene content of each region was determined using reference genome built Btau UMD3.1 (see Additional file 4: Table S1) and then compared with published data on selection sweeps in cattle.

**Table 9:** Genomic windows with extreme differences in CEh between BB (BB) and TC (TC) cattle

Chr	Region (Mb)	*F*_*ST*_ (Rank)	CEh BB	CEh TC	CEh_Diff	Genes	Candidates	Reference
5	56.2–56.6	0.20 (1)	4.786	2.462	−2.324	20	*INHBE, INHBC* (reproduction)	[12, 25, 26],
19	56.6–57.1	0.09 (214)	4.230	2.416	−1.813	20	*ATP5H* (fatness)	[29]
3	33.2–33.5	0.06 (796)	4.322	2.844	−1.478	8		
9	10.6–10.9	0.11 (89)	3.601	2.149	−1.451	1		
16	25.6–25.9	0.03 (1305)	3.839	2.734	−1.105	1		
24	61.1–62.1	0.13 (22)	2.945	2.004	−0.941	6	*BCL2* (immune response)	
5	56.6–57.8	0.10 (174)	3.789	2.887	−0.902	54	Adaptation traits	[12, 26]
1	132.4–132.6	0.06 (231)	3.144	2.268	−0.876	0		
21	57.4–57.7	0.07 (732)	2.878	2.033	−0.845	4		
14	35.5–37.3	0.08 (417)	1.886	2.811	0.926	10	*NCOA2* (reproduction)	[30]

These regions carry genes that are involved in bovine reproduction (*NCOA2*), immune function (*BCL2*) and fatness (*ATP5H*) (Table 9). It is also important to note that there were two separate instances where two highly functionally related proteins were identified in independent genomic regions: (1) monocarboxylate transporter coded by *SLC16A5* on BTA19 and its paralog coded by *SLC16A4* on BTA3 and (2) two subunits of the mitochondrial ATP synthase, the F1 catalytic core complex coded by *ATP5B* on BTA5 and the membrane-spanning F0 complex coded by *ATP5H* on BTA19 (see Additional file 4: Table S1).

## Discussion

### Inference of genomic relationships

It is well established that shared patterns of allele composition can be used to infer genomic relationships between individuals. Since both GRM and CRM are based on genome-wide similarity, it is not surprising to detect a close relationship between these two approaches. NCD shows merit as an alternative or complementary measure of genomic relatedness to GRM. Overall, short NCD distances reflect high co-sharing of genome-wide heterozygosity, runs of homozygosity and compositionally complex haplotypes, which may not be identified by other approaches. Collectively, these genomic features have implications for inbreeding, population structure and the identification of signatures of selection.

The quadratic relationship between NCD and GRM implies that NCD is particularly well adapted to discriminate closely related individuals. This conclusion is borne out by current and past data. Previously, we found that NCD can separate some full-sibs from half-sibs in cases where GRM cannot [7]. This observation may be consistent with that of [22] who found that a haplotype-based method uncovered Mendelian inconsistencies between second degree relatives more effectively than single-SNP approaches. Furthermore, in a recent application of the NCD method, we examined its application to a high-density sheep SNP dataset and found that it was able to differentiate two Poll Merino sire groups from each other and that Poll Merino individuals could be distinguished from Merino individuals by NCD but not by GRM [7]. In the data presented here, NCD clearly differentiated an individual with an unusual Zebu contribution in both the BB (separating an individual that is genetically more like a TC) and the TC (separating an animal that is genetically more like a BB) populations. This sensitive discrimination may reflect that NCD relies on ‘distance’, which enforces separation, versus the correlation’s use of ‘similarity’, which establishes connection.

Any measure of genomic similarity (whether correlation, CE, or other) needs to be clearly grounded in the biology of meiosis for it to provide meaningful estimates of genetic parameters. That is, it must yield the expected relationship values of ~1 for self–self pairs, ~0.5 for full-sibs and ~0.25 for half-sibs. These values are implicit (they emerge naturally) for correlation-based measures of relationships, but not for NCD. In this regard, the linear transformation that we used for CRM2 was superior to the quadratic transformation [17] of CRM1. The validation of CRM2 was apparent through the higher estimate of genetic variance, the corresponding slight reduction in missing heritability and the modest increase in accuracy of predictions of phenotype when compared to GRM and NRM.

Other authors have explored various options for optimising estimation of genomic relationships. For example, [23] found that parameter estimates may be biased if the genomic relationship coefficients are scaled differently from the pedigree-based estimates. They found that a reasonable scaling was possible by drawing on observed allele frequencies and scaling the relationship matrix such that the diagonal elements averaged 1. Scaling the diagonal elements to average 1 is the same logic that we applied when constructing CRM2 from the NCD values. This helps addressing the potential for biases in parameter estimation. In another example of new methods for genomic prediction, [9] published a method to derive relationships based on runs of homozygosity (ROH). They found that the ROH method produced more accurate GEBV than two alternative methods based on population-wide linkage disequilibrium and linkage. We hypothesize that this ROH method is related to our compression method. Shared ROH will obviously be clustered by compression, and thus, will contribute to the construction of the CRM. However, NCD also detects complex haplotypes beyond ROH. Understanding the biological meaning of ROH length and their implications for inbreeding and population bottlenecks is an active area of research [24]. However, it is currently not clear what inference can be drawn from the shared complex haplotypes that we are able to detect by NCD. The GRM is computed one SNP at a time, so it is not formally connected to haplotype structure.

### Detection of sweeps

Previously, we reported that by sliding a CE-based population-level window along genomes, we could identify haplotypes that are likely the result of recent positive selection [8]. Obvious examples are genomic regions with a high level of homozygosity that arise in response to selection for a single beneficial haplotype (i.e. a ‘hard’ selective sweep). The consequence is an increase in CE and, applied to human data, this approach identified classic signatures of selection such as European eye and skin colour, Asian hair texture and European and Masaai Kenyan lactase persistence [8].

In this study, we applied the compression-based sliding window analysis to two bovine populations. Analysis of genomic windows with the largest difference in CE between the two populations successfully identified regions with major effects on production traits in tropically adapted cattle [12]. The top ranked region contains two inhibin genes (*INHBE* and *INHBC*), which have been previously identified as associated with fertility traits in tropically adapted cattle [25]. The precise mechanism that drives the outlier behaviour of CE is not clear.

Inspection of the genome-wide profile revealed that nearly all extreme genomic regions were characterised by a greater CE in BB cattle, well above the genome-wide average CE (Fig. 5). This suggests that selection specific to the BB population may have caused this difference in CE between these two populations. However, the reduced CE observed in the TC cattle also appeared to have an effect on the identification of differences between populations. The top ranked region on *Bos taurus* chromosome 5 (BTA5) (between 56.2 and 56.5 Mb; Fig. 5; Table 9) co-located with an association signal with large effects on yearling weight, body condition score and coat colour, which was reported for tropically adapted cattle [12]. While the analytical approaches between these two results are very different (genome-wide association analysis (GWAS) versus CE), the two studies used the same populations of Australian BB and TC cattle. This prompted a comparison with genes that were identified as putatively under selection in five independent populations of indicine (BB and N’Dama) and taurine cattle (Holstein, Angus and Charolais) [26]. None of the genes that were identified in our study appeared to be under selection in either of the indicine breeds but 59 genes located on BTA5 [between 56.2 and 57.8 Mb; (see Additional file 4: Table S1] were previously identified in the Charolais breed [26]. Our results indicate that CEh recapitulates previous findings based on established metrics for detecting selection sweeps. However, it is interesting to note that many of the top ranked genomic windows based on CEh would not have been classified as outliers using *F*_ST_ and may constitute novel findings.

### Commercial application

In a commercial context, broiler chicken and dairy cattle breeding populations have small effective population sizes, high levels of inbreeding and both use genomic prediction as part of their modern breeding strategies, as discussed in [27]. We anticipate that the NCD method used here also has value in those two industries because it can sensitively discriminate the closely related individuals they make use of when developing their breeding programs. According to [28], for a given effective population size, the major drivers of genomic EBV accuracy are (1) LD between markers and QTL, (2) training set size, (3) heritability and (4) distribution of QTL effects. Therefore, in order to generalise our findings of the possible utility of our method in genomic prediction, we propose that future work should explore phenotypes with a lower heritability and different genetic architectures.

## Conclusions

NCD clusters a genome in a manner that is broadly similar to correlation-based approaches. Unlike GRM, NCD exploits patterns that are present in haplotypes and unlike ROH-based methods, NCD can identify and exploit haplotypes that have a more complex genotype composition. In this study, CRM2 has been validated by the fact that we show that it has a tendency to reduce the missing heritability and increase the phenotype accuracy for a moderately heritable complex trait in two bovine populations. The fine-grained resolution that NCD appears to possess may lend itself to situations for which the capacity to discriminate very closely related individuals is of particular value. A sliding window version of the analysis aimed at detecting sweeps identified regions caused by introgression of taurine haplotypes.

## Additional files

10.1186/s12711-015-0158-9 The UNIX scripts for recreating the toy GRM and CRM that are described in Figure 1. This file contains the UNIX scripts allowing the reader to recreate the example GRM and CRM from Figure 1.
10.1186/s12711-015-0158-9 Comparison of GRM and NCD across the full parameter space including self–self pairs. This figure illustrates the relationship between GRM and NCD across the full parameter space.
10.1186/s12711-015-0158-9 Comparison of GRM and NCD without self–self pairs and highlighting each pair with its average Zebu contribution. The clearest relationship between GRM and NCD is for pure bloodline pairs. This figure illustrates how the relationship between GRM and NCD is influenced by the breed characteristics of the pair of animals in question.
10.1186/s12711-015-0158-9 Genome regions with extreme differences in CEh between BB and TC cattle. This file identifies the genomic regions that are different between BB and TC cattle as determined by sliding window CEh.

## Footnotes

1 The oblique reference to George Orwell’s famous quote in Animal Farm follows the introduction of Cilibrasi and Vitanyi’s Clustering by Compression article.
