# Supplementary material for: Compression distance can discriminate animals by genetic profile, build relationship matrices and estimate breeding values
Source: Genet Sel Evol. 2015 Oct 13;47:78. doi: 10.1186/s12711-015-0158-9 (PMC4604992; doi:10.1186/s12711-015-0158-9)
Supplement: Supplementary file 1 — 10.1186/s12711-015-0158-9 The UNIX scripts for recreating the toy GRM and CRM that are described in Figure 1. This file contains the UNIX scripts allowing the reader to recreate the example GRM and CRM from Figure 1. [file 12711_2015_158_MOESM1_ESM.docx]

# Supplementary File

# Compression distance can discriminate animals by genetic profile, build relationship matrices and estimate breeding values.

# N.J. Hudson, L.R. Porto-Neto, J.W. Kijas and A. Reverter.

# Genetic Selection Evolution.

The following UNIX script is intended to perform the required operations to compute compression efficiency (CE), normalized compression distance (NCD) and the three compression-based relationship matrices (CRM1, CRM2 and CRM3) using the toy example of Figure 1 comprising five individuals and 30 SNP genotypes. It assumes the existence of a genotype file named “5genos.txt” with exactly the following content (cut&paste and save with the name “5genos.txt’ in the same folder where the main script will be run):

02000

02001

02002

00110

00111

00122

00220

00221

00212

00000

10001

10002

10110

11111

11122

11220

11221

11212

11000

11001

21002

21110

21111

22122

22220

22221

22212

22000

22111

22222

It also requires the presence of two AWK scripts, one to perform the transpose function (“transpose.awk”), and one to compute the correlation coefficient (“corr.awk”).

The file “transpose.awk” is as follows (cut&paste and save with the name “transpose.awk” in the same folder where the main script will be run):

######## SCRIPT BEGINGS HERE #########

BEGIN {FS=" "}{

for (i=1;i<=NF;i++){

arr[NR,i]=$i;

if(big <= NF)

big=NF;

}

}

END {

for (i=1;i<=big;i++){

for (j=1;j<=NR;j++){

printf("%s%s",arr[j,i],OFS);

}

printf("\n");

}

}

######## SCRIPT FINISHES HERE #########

The file “corr.awk” is as follows (cut&paste and save with the name “corr.awk” in the same folder where the main script will be run):

######## SCRIPT BEGINGS HERE #########

{ v1[NR]=$1; v2[NR]=$2}

END{ for(i=1;i<=NR;i++){

s1 += v1[i]; ss1 += v1[i]*v1[i]

s2 += v2[i]; ss2 += v2[i]*v2[i]

ss12 += v1[i]*v2[i] }

mean1 = s1/NR

var1 = ( ss1 - (s1*s1)/NR ) / (NR-1)

mean2 = s2/NR

var2 = ( ss2 - (s2*s2)/NR ) / (NR-1)

num = ( ss12 - (s1*s2)/NR ) / (NR-1)

den = sqrt(var1 * var2)

corr = num / den

printf"%12.5f\n",corr

}

######## SCRIPT FINISHES HERE #########

Upon execution, the following 4 files are created:

1. Compress.out: heterozygocity and compression efficiency (CE) for each individual.
2. NCD.out: normalized compression distance for every pair of individuals.
3. CEMatrix_4CRM3.out: The (4 windows x 5 individuals) matrix with the CE values needed for the computation of CRM3.
4. NCD_CRM123.out: The NCD and CRM1, CRM2 and CRM3 for every pair of individuals. This file makes NCD.out redundant.

The main script starts below. This should be cut&paste into a single file and store it in the same folder under the arbitrary name of “mainscript.sh” and execute it using the following command “sh mainscript.sh”

################ MAIN SCRIPT BEGINGS HERE ###############

##################################################

# Remove these 3 output files in case they exists

# because they will be appended at each iteration

##################################################

rm –f Compress.out NCD.out CEMatrix_4CRM3.out

genofile=5genos.txt

N=1

MN=5

while [ $N -le $MN ]

do

awk -v N=$N '{print substr($1,N,1)}' $genofile > anim1.gen.j

ntot1=`awk '{print $1}' anim1.gen.j | wc -l | awk '{print $1}'`

het=`awk '$1==1 {print $1}' anim1.gen.j | wc -l | \

awk -v ntot1=$ntot1 '{print $1/ntot1}'`

################################################

# Assuming the 6^th^ column out of ‘ls –l’ command

# is the one containing the size of the file.

# It could be the 5^th^ column depending on your

# UNIX flavour. Check first.

################################################

bef=`ls -l anim1.gen.j | awk '{print $6}'`

gzip anim1.gen.j

Zx=`ls -l anim1.gen.j.gz | awk '{print $6}'`

CE=`echo $bef $Zx | awk '{print ($1-$2)/$1}'`

echo $N $het $CE >> Compress.out

gunzip anim1.gen.j.gz

M=`expr $N`

MM=`expr $MN`

while [ $M -le $MM ]

do

awk -v M=$M '{print substr($1,M,1)}' $genofile > anim2.gen.j

gzip anim2.gen.j

Zy=`ls -l anim2.gen.j.gz | awk '{print $6}'`

gunzip anim2.gen.j.gz

paste -d " " anim1.gen.j anim2.gen.j > anim12.gen.j

gzip anim12.gen.j

Zxy=`ls -l anim12.gen.j.gz | awk '{print $6}'`

gunzip anim12.gen.j.gz

NCD=`echo $Zx $Zy $Zxy | \

awk '{print ($3-($1<$2?$1:$2))/($1>$2?$1:$2)}'`

echo $N $M $NCD >> NCD.out

rm anim2.gen.j anim12.gen.j

M=`expr $M + 1`

done

rm anim1.gen.j

N=`expr $N + 1`

done

################################

# Build CRM1 and CRM2 in one go

################################

SelfSelf=`awk ‘$1==$2 {sum+=$3; n++} END {print sum/n}’ NCD.out`

NoSelfMin=`awk ‘$1!=$2 {print $3}’ NCD.out | sort –k1g | head -1`

NoSelfMax=`awk ‘$1!=$2 {print $3}’ NCD.out | sort –k1gr | head -1`

awk –v ss=$SelfSelf –v nsmi=$NoSelfMin –v nsma=$NoSelfMax \

'{print $0, 2.5*exp(-5*$3), \

($1==$2?ss/$3:0.75*(1-(($3-nsmi)/(nsma-nsmi))))}' \

NCD.out > crm1crm2.j

####################################################################

# Build CRM3:

#

# Note that it takes 32 bytes just to create the internal signature

# of gzip on a file with a name 11 characters in length such as

# “anim1.gen.j”. This is of relevance when computing CE for really

# small files (such as the ones in this toy example).

#

# Create 4 files of 7, 7, 7, and 9 SNP genotypes each and compute

# the pair-wise correlation among the resulting CE.

# There are more intelligent ways to create these four “ggg” files.

# This is brute force.

####################################################################

awk 'NR<=7 {print $0}' $genofile > ggg.1

awk 'NR>7 && NR<=14 {print $0}' $genofile > ggg.2

awk 'NR>14 && NR<=21 {print $0}' $genofile > ggg.3

awk 'NR>21 {print $0}' $genofile > ggg.4

for window in 1 2 3 4

do

N=1

MN=5

rm crm3.j

while [ $N -le $MN ]

do

awk -v N=$N '{print substr($1,N,1)}' ggg.$window > anim1.gen.j

bef=`ls -l anim1.gen.j | awk '{print $6}'`

gzip anim1.gen.j

Zx=`ls -l anim1.gen.j.gz | awk '{print $6-32}'`

CE=`echo $bef $Zx | awk '{print ($1-$2)/$1}'`

echo $CE >> crm3.j

rm anim1.gen.j.gz

N=`expr $N + 1`

done

awk -f transpose.awk crm3.j >> CEMatrix_4CRM3.out

done

rm ggg.? crm3.j

N=1

MN=5

while [ $N -le $MN ]

do

M=`expr $N`

MM=`expr $MN`

while [ $M -le $MM ]

do

echo $N $M

awk -v N=$N -v M=$M '{print $N, $M}' CEMatrix_4CRM3.out | \

awk -f corr.awk >> crm3.j

M=`expr $M + 1`

done

N=`expr $N + 1`

done

paste -d " " crm1crm2.j crm3.j > NCD_CRM123.out

rm crm1crm2.j crm3.j
