# Supplementary figures and images for: Compression distance can discriminate animals by genetic profile, build relationship matrices and estimate breeding values
Source: Genet Sel Evol. 2015 Oct 13;47:78. doi: 10.1186/s12711-015-0158-9 (PMC4604992; doi:10.1186/s12711-015-0158-9)

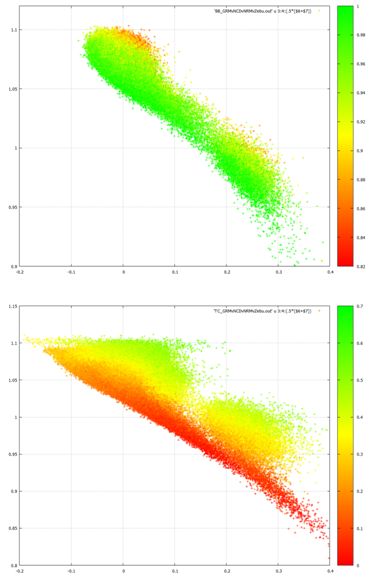

Supplement: Supplementary file 3 — 10.1186/s12711-015-0158-9 Comparison of GRM and NCD without self–self pairs and highlighting each pair with its average Zebu contribution. The clearest relationship between GRM and NCD is for pure bloodline pairs. This figure illustrates how the relationship between GRM and NCD is influenced by the breed characteristics of the pair of animals in question. [file 12711_2015_158_MOESM3_ESM.jpeg]
